# Uremic stomatitis^☆^

**DOI:** 10.1016/j.abd.2020.09.019

**Published:** 2022-03-17

**Authors:** Paulo Ricardo Martins Souza, Gabriela Mosena, Manuela Lima Dantas, Gerson Vettorato

**Affiliations:** a Dermatology Service, Santa Casa de Misericórdia de Porto Alegre, Porto Alegre, RS, Brazil; b Department of Dermatology, Universidade Federal de Ciências da Saúde de Porto Alegre, Porto Alegre, RS, Brazil; c Clínica Privada de Dermatologia, Caxias do Sul, RS, Brazil; d Clínica Privada de Dermatologia, Porto Alegre, RS, Brazil

Dear Editor,

A 42-year-old male patient was seen at the Dermatology Service due to the presence of whitish lesions on the oral mucosa, affecting mainly the tongue. Moreover, he reported significant dysgeusia and a lack of appetite. He had chronic kidney disease and had been undergoing conservative treatment so far. The examination of the oral cavity showed whitish plaques with threadlike projections adhered to the lateral borders of the tongue (Figure 1, Figure 2) and a white plaque on the left cheek mucosa (Fig. 3). He had ketone breath on examination. The patient was awaiting dialysis and had a serum creatinine level of 17 mg/dL, with uremia of 200 mg/dL. After a few hemodialysis sessions, the lesions regressed significantly.

**Figure 1:**
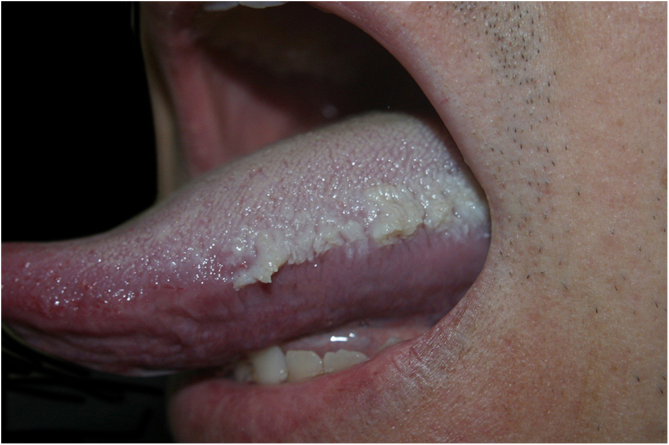
Whitish plaque with threadlike projections adhered to the left lateral border of the tongue.

**Figure 2:**
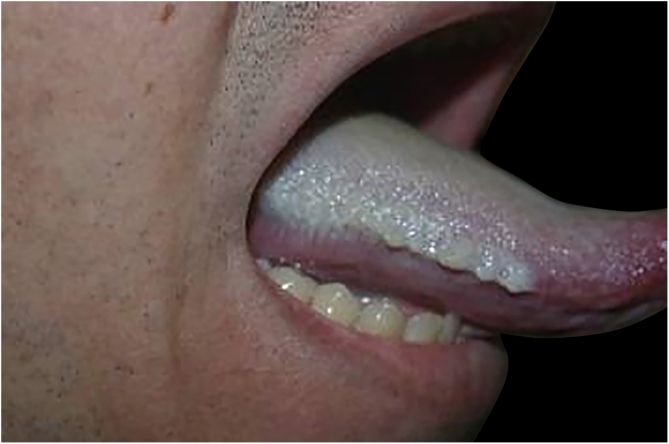
Whitish plaque with threadlike projections adhered to the right lateral border of the tongue.

**Figure 3:**
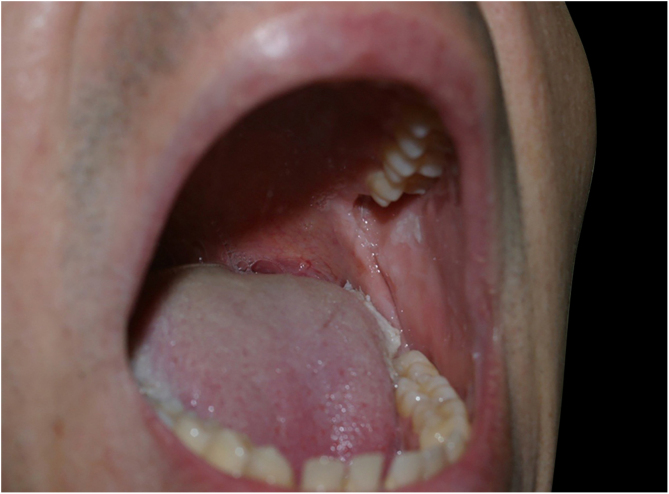
Whitish plaque on the left cheek mucosa.

Uremic stomatitis is an underreported disease of the oral mucosa, possibly associated with long-term uremia in patients with chronic kidney disease.^1^ It was first mentioned by Lancereaux in 1887 and described by Barie in 1889 as an uncommon but characteristic complication of advanced kidney disease.^2^ It has a low incidence^2^ which notably decreased with the advent of dialysis, and is rarely seen nowadays.^3^ The etiology remains unknown, and it has been suggested that it may be due to high levels of ammonia compounds.^1^ Ammonia is formed through the action of bacterial ureases that modify salivary urea, which is elevated in renal patients. The clinical characteristics are poorly defined and are rarely detailed in publications.^1^ The affected patients may complain of pain, dysgeusia, and a burning sensation.^1^, ^4^ Four clinical types of uremic stomatitis have been described: pseudomembranous, ulcerative, hemorrhagic, and hyperkeratotic.^2^ The ulcerative type is the most common,^2^ with an erythematous appearance, and the hyperkeratotic type is a rare alteration that can occur in long-term renal failure. Diagnosis is based on clinical signs and symptoms, and histopathology is characterized by epithelial hyperplasia and unusual hyperparakeratinization.^1^, ^5^ Lichen planus, hypertrophic candidiasis, oral hairy leukoplakia, and vitamin deficiencies are important differential diagnoses.^4^ The treatment consists in improving blood urea levels.^2^ The manifestations usually persist for two to three weeks. Hydrogen peroxide washes can contribute to the elimination of anaerobic bacteria that produce ammonia.^1^ Despite the high frequency of patients with kidney disease, only a few cases of uremic stomatitis have been published. Investigations are required for a better understanding of the pathogenic mechanism of this disorder.

## Financial support

None declared.

## Authors' contributions

Paulo Ricardo Martins Souza: Approval of the final version of the manuscript; drafting and editing of the manuscript; intellectual participation in the propaedeutic and/or therapeutic conduct of the studied cases; critical review of the literature; critical review of the manuscript.

Gabriela Mosena: Approval of the final version of the manuscript; drafting and editing of the manuscript; critical review of the literature; critical review of the manuscript.

Manuela Lima Dantas: Approval of the final version of the manuscript; drafting and editing of the manuscript; critical review of the literature; critical review of the manuscript.

Gerson Vettorato: Approval of the final version of the manuscript; drafting and editing of the manuscript; intellectual participation in the propaedeutic and/or therapeutic conduct of the studied cases; critical review of the literature; critical review of the manuscript.

## Conflicts of interest

None declared.
